# Spontaneous perspective-taking in real-time language comprehension: evidence from eye-movements and grain of coordination

**DOI:** 10.1038/s41598-024-58699-z

**Published:** 2024-04-05

**Authors:** Yipu Wei, Yingjia Wan, Michael K. Tanenhaus

**Affiliations:** 1 School of Chinese as a Second Language, Peking University, Beijing, 100871 China; 2 CAS Key Laboratory of Behavioral Science, Institute of Psychology, Chinese Academy of Sciences, Beijing, 100101 China; 3 Department of Psychology, University of Chinese Academy of Sciences, Beijing, 101408 China; 4 Department of Brain and Cognitive Sciences, University of Rochester, Rochester, NY 14627 USA

**Keywords:** Human behaviour, Psychology

## Abstract

Linguistic communication requires interlocutors to consider differences in each other’s knowledge (perspective-taking). However, perspective-taking might either be spontaneous or strategic. We monitored listeners’ eye movements in a referential communication task. A virtual speaker gave temporally ambiguous instructions with scalar adjectives (“big” in “big cubic block”). Scalar adjectives assume a contrasting object (a small cubic block). We manipulated whether the contrasting object (a small triangle) for a competitor object (a big triangle) was in common ground (visible to both speaker and listener) or was occluded so it was in the listener’s privileged ground, in which case perspective-taking would allow earlier reference-resolution. We used a complex visual context with multiple objects, making strategic perspective-taking unlikely when all objects are in the listener’s referential domain. A turn-taking, puzzle-solving task manipulated whether participants could anticipate a more restricted referential domain. Pieces were either confined to a small area (requiring fine-grained coordination) or distributed across spatially distinct regions (requiring only coarse-grained coordination). Results strongly supported spontaneous perspective-taking: Although comprehension was less time-locked in the coarse-grained condition, participants in both conditions used perspective information to identify the target referent earlier when the competitor contrast was in privileged ground, even when participants believed instructions were computer-generated.

## Introduction

Perspective-taking refers to the ability to mentalize others’ viewpoints that are distinct from one’s own. This may involve reasoning about others’ knowledge on object existence in false-belief tasks^1,2^, giving descriptions aligned with others’ spatial perspectives^3^, and orienting attention to the direction of others’ gaze as revealed in visual cognition studies^4,5^.

Perspective-taking is essential for linguistic communication. Conversation, the primary arena of language use, requires participants to monitor differences in each other’s knowledge, which includes distinguishing between information that is privileged to one participant (private or privileged ground), and information that is shared (common ground). Talkers typically ask questions about information that is unknown to themselves but likely known to their addressee; conversely, talkers often make assertions about information that is shared or privileged to them^6^. The form of numerous linguistic expressions is conditioned on differences in the strength of evidence that interlocutors have for the information in an utterance^7^. Moreover, generating and interpreting referring expressions, one of the foundations of communication, requires speakers and listeners to distinguish between shared and privileged knowledge. A speaker will use a longer referential expression, such as “my son-in law, Charlie” versus the shorter “Charlie”, when she assumes the listener needs that additional information to identify their intended referent. Conversely, with the shorter expression, the listener, who likely knows multiple Charlie’s, must identify the intended referent as the mutually known Charlie who is relevant to the given context.

Although perspective-taking is necessary for interpreting referring expressions, it remains controversial, under what conditions, and especially, when in processing, listeners make use of perspective differences. In a pioneering study, Keysar et al.^8^ monitored eye movements in a referential communication task. A confederate speaker instructed a naïve participant (listener) to move objects in a 16-box cubby. “Visual co-presence” was manipulated to create perspective differences^9^: The speaker could not see some occluded objects, which were therefore in the listener’s privileged ground. On critical trials, the referring expression (e.g., “the tape”) was a better fit to an object in privileged ground (a roll of sticky tape) than the object in common ground (a cassette tape). Listeners’ initial eye-movements were more likely to be directed to the privileged-ground object. This finding rules out models of reference resolution in which listeners only consider objects in common ground, but, as Keysar et al. acknowledge, it is also consistent with constraint-based models^10^. Keysar et al.^8,11^ argued that because perspective-taking is resource-demanding, listeners’ initial processing is *egocentric* and possible perspective differences are considered only when miscommunication or ambiguity arises which requires use of perspective.

Subsequent research found evidence for immediate perspective-taking^6,12^, using temporarily ambiguous instructions, which eliminates the need to have privileged-ground objects that are a better referential fit to the instruction, and the requirement that listeners use perspective to disambiguate the referent. However, these studies used small referential domains, typically four or five objects. Therefore, the results are compatible with a view that with small, restricted, referential domains, listeners can strategically consider perspective differences, as opposed to perspective being a constraint that is spontaneously used in the earliest moments of processing.

### Overview

The current study adjudicated between spontaneous and strategic perspective-taking by monitoring eye movements in a referential communication task with a larger set of potential referents, making strategic perspective-taking more costly. Linguistic instructions contained temporarily ambiguous referring expressions where use of perspective wasn’t required for identifying the referent but would allow for earlier identification of the referent. The timing of fixations to the referent was the primary dependent measure and was used to determine whether listeners were considering perspective differences. We used two manipulations to encourage or discourage strategic perspective-taking. First, and most importantly, we preceded the referential communication task with a joint puzzle-solving task, with the same partner, in which we manipulated the grain of coordination. In the fine-grained coordination condition, the placement of the virtual partner’s piece biased participants to focus on a small number of relevant objects, which should encourage strategic perspective-taking in the subsequent referential communication. The coarse-grained condition did not restrict the referential domain because there was no contingency between the partners’ placement of pieces. We also manipulated whether the participants believed their partner was a computer or a human, reasoning that strategic perspective-taking would be more likely with human partners.

### Logic of the referential communication task

We followed Heller et al.^12^ in using instructions with pre-nominal scalar adjectives, but with Mandarin Chinese. When hearing a scalar adjective such as “big” (*dàde* in Chinese), listeners assume that a big object and its smaller contrast object are both visually accessible to the speaker. If there are two similar-sized blocks, a cubic one and a triangular one, but only the cubic block has a size contrast (a smaller cubic block), shortly after listeners hear “big”, gaze shifts to the bigger cubic block, with some looks to the contrasting, smaller cube^13^. If, however, both the (target) cubic and the (competitor) triangle blocks have visible size contrasts, gaze does not shift to the referent until after the listener hears “cubic”^14^. We manipulated whether the competitor contrast (a small triangle) was in the common ground (visible to both speaker and listener) or in the listener’s privileged ground (occluded from the speaker), in which case it would be infelicitous for the speaker to refer to the competitor using the scalar adjective “big”. In the privileged ground condition, perspective-taking would allow the listener to resolve the target referent earlier. Because temporary ambiguity is ubiquitous as utterances unfold, there is no demand for listeners to strategically use perspective to successfully determine the referent: Heller et al.^12^ found clear evidence for immediate perspective-taking with a confederate speaker and a display containing only four objects.

If perspective-taking is strategic, it is likely to occur primarily in circumstances with a small referential domain where participants can selectively focus on a small set of objects, and is correspondingly unlikely in complex visual displays with more objects, which would be difficult to maintain in working memory. Therefore we used larger sets of objects in the visual display. In fact, recent studies suggest that eye-movements in visual world paradigms are less time-locked with larger displays, making it plausible that strategic perspective-taking would be unlikely with more objects in the display^15,16^.

### Non-linguistic coordination manipulation

Our coordination manipulation was inspired by recent work on how participation in a joint activity influences non-linguistic, prosocial behaviors^17,18^, including aspects of generosity and perspective-taking in children, such as choosing an appropriate gift for a partner^19^. Crucially, grain of coordination is important, even when the overall goal of the joint activity is the same^17^. We hypothesized that reference resolution would be more time-locked in the finer-grained coordination condition. We reasoned that, if perspective-taking is strategic, it should interact with grain of coordination.

We created a situation in which the finer-grained coordination should bias participants to focus on a more restricted set of referents. A participant and a (simulated) partner took turns placing pieces to complete a puzzle in a screen-based task. In the *finer-grained* condition, pieces assigned to the players, once placed, were immediately adjacent to each other, thus creating a highly interdependent coordinative experience in which the participant expects their partner to focus on the same restricted referential domain. In the *coarser-grained* condition, players’ pieces were placed adjacent to the previous piece they placed but far away from the other player’s last-placed piece, leading to a more independent collaborative experience.

This manipulation was designed to modulate participants’ referential domain. As interlocutors are constantly monitoring what their partners are attending to in a visual workspace during a joint task^20^, the referential domains of interlocutors in visual contexts could be entrained through joint experience. For instance, interlocutors’ eye movements are coupled during conversation^21^ and their referential domains become aligned^22^. Our manipulation biased participants to either consider a more or less-restricted referential domain. We reasoned that, if perspective-taking is strategic, it would be more likely to occur when the participant was encouraged to consider a restricted referential domain, making it comparable to previous studies with a smaller set of objects.

There is, however, an alternative possibility. Past research has shown that people in close social relations (e.g., friends, spouses, etc.) are less likely to take each other’s perspectives than strangers^23^, which suggests that people adjust their perspective-taking tendencies based on motivation and needs. It is possible, then, that close coordination may reduce perspective-taking by increasing social closeness which reduces the necessity of perspective-taking and biases people towards overestimating similarities. Even if this were the case, it would predict that perspective-taking would interact with grain of coordination, which would provide evidence for strategic perspective-taking.

In sum, an interaction between early perspective-taking and grain of coordination (e.g., perspective-taking only found in the fine-grained coordination as the referential domain account predicts, or only in the coarse-grained coordination in line with the social closeness prediction) would provide evidence that perspective-taking is strategic.

### Partner type manipulation

We also manipulated whether participants believed their partner was a pre-programmed computer or another person. The partner in the puzzle game served as the speaker in the referential communication task. Participants in those tasks could be sensitive to whether tasks are interactive^24,25^ and whether the partner is a confederate^26^. If perspective-taking is strategic, participants might be less inclined to take perspective when they believe they are interacting with a pre-programmed computer partner, as they would be less motivated and find it unnecessary. Indeed, spatial perspective-taking tasks have found more egocentric behavior in describing and interpreting spatial directions when the partner is a computer^27,28^. If, however, perspective-taking is automatic and spontaneous, participants should consider perspective differences regardless of partner type.

### Summary

By increasing the size of the referential domain and adopting the novel approach of a non-linguistic coordination manipulation, we explored the generality of spontaneous/automatic perspective-taking in referential communication. If spontaneous perspective-taking is the general case, we should find effects of perspective-taking, regardless of grain of coordination and beliefs about the type of partner as indexed by an increase in eye movements towards the target prior to receiving disambiguating linguistic details (i.e., the shape adjective). If, however, perspective-taking requires strategic allocation of resources, we would either not see any effects of perspective-taking, see effects only in the fine-grained coordination condition, or perhaps only with a combination of fine-grained coordination and belief that the partner was a person.

## Results

### Overall results

We conducted a multilevel logistic regression analysis of fixation proportions in the referential communication task during the critical speech interval (from the onset of the scalar adjective until the revealing of shape information, see details in “Methods” section). We also report a window-based analysis which evaluated mean fixation proportions of different conditions in three temporal windows (see Supplementary—Window-based Analysis). Both analyses find effects of ground across conditions, emerging immediately after the scalar adjectives and before the shape information was uttered in the speech. Such ground effects, which demonstrate sensitivity to perspective differences, were robust in both fine-grained and coarse-grained coordination conditions and regardless of partner type.

### Model selection

We evaluated proportion of looks to different areas of interest as function of ground (shared vs. privileged), coordination (fine-grained vs. coarse-grained) and partner type (computer vs. human). We also examined whether these effects interacted with the variable of time, which was included in the model to simulate how they changed over time.

Including partner type did not increase the model fit (Target: χ^2^ = 0.03, *df* = 1, p = 0.87; Target-set: χ^2^ = 0.07, *df* = 1, p = 0.79), which indicated that proportions of looks to the target and target-set were not regulated by partner type. Therefore, we collapsed across partner type in the analyses to increase statistical power.

The three-way interaction of time, ground and coordination did not change the model fit in the target analysis (χ^2^ = 0.36, *df* = 1, *p* = 0.55). Therefore, the final model only includes the three factors and their two-way interactions. In the target-set analysis, however, the three-way interaction of time, ground and coordination significantly improved the model fit (χ^2^ = 20.36, *df* = 1, *p* < 0.001) and therefore was included in the final equation to model looks to the target-set.

### Ground effects: comparing proportion of looks in privileged vs shared ground

**Figure 1:**
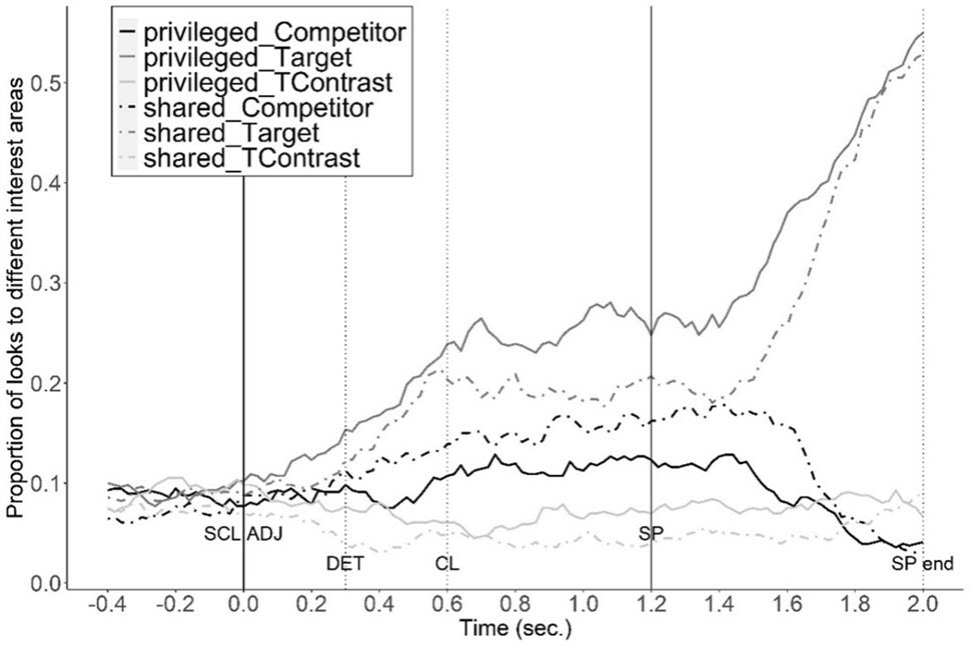
Proportion of looks to the target, competitor and target-contrast under the privileged-ground condition and the shared-ground condition. Time interval: from 0.4 s before the onset of scalar adjective (SCL ADJ) to 0.2 s after the average offset of the shape noun phrase (SP end). The onsets of scalar adjective (SCL ADJ), the determiner (DET), the classifier (CL) and the shape (SP) are marked with vertical lines (same for figures below).

When both big objects have a size contrast in common ground, the earliest point that reference could be established is when the participant hears the shape. However, when only the target has a size contrast in common ground, perspective-taking would allow disambiguation at the scalar adjective. Figure 1 presents the proportion of looks to the target, competitor and target-contrast from 400 ms before the onset of the scalar to the end of shape information. The ambiguous region (the critical region) began 200 ms after the scalar onset and ended 200 ms after the onset of the shape adjective (200 ms is the earliest point in time when signal-driven fixations occur in Visual World studies^29^). The ground effect was assessed by: (i) looks to the target object; (ii) comparison between the looks to the competitor and target-contrast; and (iii) looks to the target-set (target and target-contrast) during the critical region.

When the competitor-contrast was in privileged ground, the proportion of target looks increased earlier compared to when it was in common ground, resulting in a significant difference in looks to the target between the privileged and shared-ground conditions in both the fine-grained coordination (β = 0.22, *SE* = 0.03, *z* = 7.79, *p* < 0.001) and coarse-grained coordination conditions (β = 0.44, *SE* = 0.03, *z* = 15.24, *p* < 0.001).

Looks to the target-contrast compared to the size competitor also revealed ground effects. Ground and IA (interest area) interacted: in shared-ground conditions, there were fewer looks to the target-contrast compared to the competitor (β =  − 1.35, SE = 0.03, z =  − 43.05, p < 0.001); in privileged-ground conditions, there were more looks to the target-contrast, compared to shared-ground conditions (β = 0.84, SE = 0.04, z = 19.81, p < 0.001). Because looks to the target and its size contrast both reflect processes associated with identifying the referent of an expression with scalar contrast, we combined looks to the target and target-contrast into a target-set. The proportion of looks to the target-set was significantly higher in the privileged-ground condition (vs. shared-ground condition, both in the fine-grained coordination (β = 0.46, *SE* = 0.03, *z* = 17.15, *p* < 0.001) and the coarse-grained coordination conditions (β = 0.38, *SE* = 0.03, *z* = 14.495, *p* < 0.001).

**Figure 2:**
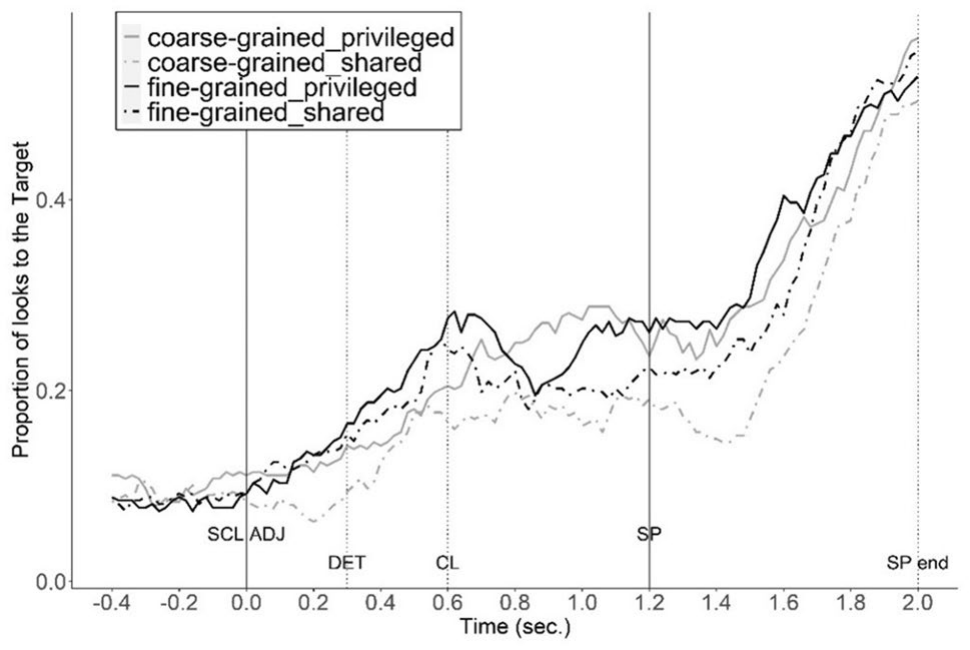
Proportion of looks to the target in different ground and coordination conditions. Time interval: from 0.4 s before the onset of scalar adjective (SCL ADJ) to 0.2 s after the average offset of the shape noun phrase (SP end).

**Figure 3:**
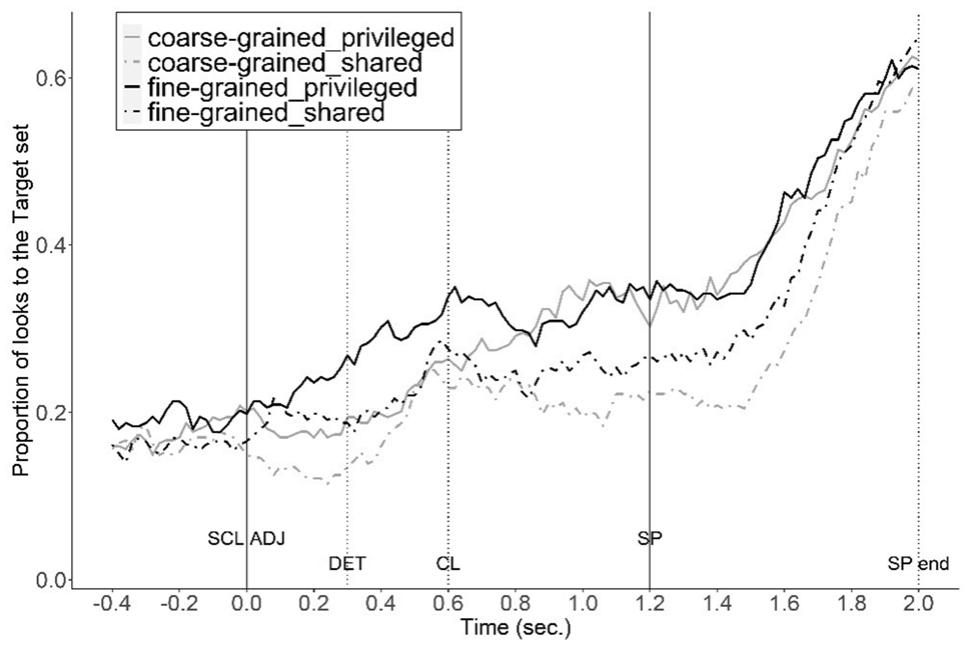
Proportion of looks to the target-set (target and target-contrast) in different ground and coordination conditions. Time interval: from 0.4 s before the onset of scalar adjective (SCL ADJ) to 0.2 s after the average offset of the shape noun phrase (SP end).

Figures 2 and 3 show the proportion of fixations on the target and target-set in the different ground and coordination conditions.

In addition to the ground effects observed in both coordination groups, the fine-grained coordination group fixated more on the target and the target-set compared to the coarse-grained coordination group (Target: β = 0.32, *SE* = 0.14, *z* = 2.26, *p* = 0.02; Target-set: β = 0.27, *SE* = 0.13, *z* = 2.07, *p* = 0.04). However, there was no evidence that fine-grained coordination increased the ground effect. In fact, the difference in looks to the target between shared-ground and privileged-ground is smaller in the fine-grained coordination group compared to the coarse-grained coordination group (Target: β = − 0.22, *SE* = 0.04, *z* = − 5.49, *p* < 0.001; Target-set: β = − 0.08, *SE* = 0.04, *z* = − 2.13, *p* = 0.03). This is due to faster reference resolution for the fine-grained coordination group, as indicated by an earlier peak of fixation proportions to the target (around 0.6s, see the black line in Figs. 2, 3), which drops within the critical time window, a common pattern once reference resolution is established.

### Changes of looks over time

Changes in proportion fixations over time differed in the privileged ground and the shared ground, as indicated by an interaction between time and ground (Target: β = 0.25, *SE* = 0.06,* z* = 4.41, *p* < 0.001; Target-set: β = 0.49, *SE* = 0.08, *z* = 6.40, *p* < 0.001). Significant interactions of ground and time arise because looks to the target and target-set increase earlier in the privileged-ground condition, compared to the shared-ground condition.

The interaction of time and coordination in the privileged-ground conditions shows that the proportion of looks to the target in the fine-grained coordination groups peaked earlier compared to the coarse-grained coordination group (β = − 0.25, SE = 0.06, z = − 4.34, p < 0.001). Participants in the fine-grained coordination group had more time-locked interpretation of referents.

There was also a significant interaction effect of ground, coordination, and time in target-set analysis (β =  − 0.48, *SE* = 0.11, *z* =  − 4.51, *p* < 0.001). Pair-wise comparisons among the four conditions show that, in the shared-ground conditions, changes of looks to the target-set do not differ between the fine-grained and coarse-grained coordination groups (β =  − 0.04, *SE* = 0.08, *z* =  − 0.51, *p* = 0.61). However, in the privileged-ground conditions, the change in looks in the fine-grained coordination group is significantly different from the coarse-grained coordination group (β =  − 0.55, *SE* = 0.07, *z* =  − 7.41, *p* < 0.001). As shown in Fig. 3, the proportion of looks to the target-set in privileged-ground condition diverges from that in the shared-ground condition and peaks earlier in the fine-grained coordination group compared to the coarse-grained coordination group. Thus, as predicted, experience with fine-grained coordination in a non-linguistic task led to more time-locked comprehension.

## Discussion

The primary question addressed in this study was whether real-time perspective-taking in reference resolution is strategic or spontaneous in referential communication tasks where visual co-presence creates perspective differences. Previous studies established that listeners can use perspective-taking during the earliest moments of reference resolution, however, these results were compatible with two alternatives. The first is that perspective-taking is spontaneous. The second is that listeners can strategically consider differences in perspective when the referential domain is restricted to a small set of objects.

We assessed perspective-taking by monitoring eye-movements in a referential communication task using a display with a larger set of objects than used in previous studies that found evidence for early perspective-taking. We used instructions containing scalar adjectives, e.g., “big cubic” which convey that there is another potential (contrast) referent that only differs from the intended referent (a big cubic shape) on the dimension mentioned by the scalar adjective (a small cubic shape). We compared a condition where a competitor shape also had a contrast competitor in common ground with a condition where the size contrast for the competitor was in the listener’s privileged ground. When both size contrasts were in common ground, the referent could not be identified until the listener heard the shape name, whereas when the competitor contrast was in privileged ground, perspective-taking would allow the listener to identify the intended referent at the scalar adjective. In addition to using a larger number of objects than in previous studies of perspective-taking, we used pre-recorded instructions to eliminate any potential bias from use of a confederate.

We used a novel non-linguistic manipulation to assess whether perspective-taking is strategic. In a turn-taking puzzle task, motivated by the literature on how grain of coordination affects prosocial behavior, the players either added pieces to the same small region of the puzzle, creating closer collaboration between the participant and the partner (fine-grained coordination) or to a spatially distinct area (coarse-grained coordination).

The results were unequivocal. Ground effects, which demonstrate listeners’ awareness of their partners’ perspectives in referential communication, were observed for both partner types and both coordination conditions. Listeners clearly considered the differences in what objects they could see but the speaker could not. This pattern held regardless of whether the participant believed they were interacting with a computer or a human and whether the listener could anticipate that the referential domain would be restricted. Although the condition with coarse-grained coordination resulted in less time-locked comprehension, it did not reduce perspective-taking. Compared to the coarse-grained coordination, fine-grained coordination did not lead participants to perform better in perspective-taking as the strategic perspective-taking account would predict. Moreover, it did not decrease the scale of perspective-taking effects as one would expect if closer relations between communicators reduces the need and motivation for perspective-taking. We found no evidence for strategic perspective-taking. Both coarse-grained and find-grained coordination groups exhibited early perspective-taking: When the competitor contrast was in the privileged ground, reference resolution, as indexed by eye-movements to the intended referent, preceded the mention of the shape in the instruction. Ground effects were observed in both partner types, demonstrating sensitivity to perspective differences regardless of whether the partner is perceived as human or computer. This observation aligns with prior studies that have shown similar levels of perspective-taking abilities with both human and robotic partners^30^.

Our findings provide strong evidence for the view that perspective-taking is the default in real-time referential communication, at least when perspective differences are manipulated by visual co-presence. This result is expected under constraint-based models of comprehension in which perspective-taking, along with other pragmatic information, is understood as a probabilistic constraint used in the earliest moments of language comprehension^10^.

This class of model also accounts for results which have been interpreted as evidence for egocentrism, for example, Keysar et al.^8,11^). In those studies, the object in privileged ground was a better referential fit for the object in privileged ground. A listener who uses perspective to help interpret a referential expression will, of course, be aware of objects that are in her privileged ground. Thus, it is not surprising that attention will be drawn to a privileged ground object when it is a better referential fit for a speaker’s referring expression.

It is important, however, to acknowledge that we examined perspective-taking under a specific set of conditions. We used scalar adjectives and manipulated whether a potential referent was in common or privileged ground using physical co-presence. In subsequent research it will be important to explore the limits of spontaneous perspective-taking.

Another important contribution of this study is that finer-grained coordination in a non-linguistic task resulted in more time-locked comprehension. This is consistent with previous findings on positive social effects of fine-grained, highly interdependent coordination^17,18^. In the fine-grained coordination condition, since the players’ pieces were immediately adjacent to each other, the feeling of interdependence and cooperation should be stronger for the fine-grained coordination group, which may lead to more attention to the partner’s utterances and therefore more time-locked comprehension. This interpretation is supported by the results of a post-test questionnaire measuring participants’ feelings of cooperation (Supplementary—Post-test Questionnaire). To the best of our knowledge, this is the first demonstration that grain of coordination in a non-linguistic task affects subsequent real-time language processing. It is important to note that both the puzzle and referential communication tasks in this study had a strong spatial component. In future research, it will be important to see whether coordination effects will generalize to non-spatial tasks. Although the grain of coordination did not affect perspective-taking, it might well affect comprehension. For example, more time-locked comprehension might result in listeners detecting and correcting potential miscommunication more quickly. It might also affect the timing of back-channel information, such as expressions of understanding (uh-huh) or non-linguistic cues such as nods that help signal whether a listener is understanding her interlocutor. Fine-grained coordination in a non-linguistic domain might also increase sensitivity to pragmatic information such as the general cooperativeness of the speaker. These questions could be fruitful topics for further research.

Our results with scalar adjectives provide a baseline for exploring possible effects of grain of coordination on linguistic perspective-taking with other structures. In future research it will be important to examine the effects of coordination on types of pragmatic inference that have greater speaker variability in their use compared with scalar adjectives. We speculate that coordination effects might be stronger when the pragmatic priors for a linguistic construction are weaker. With structures where perspective-taking is less automatic, stronger effects of grain of coordination might emerge.

In sum, the results of previous studies finding immediate perspective-taking were consistent with the hypothesis that listeners can strategically use perspective with highly circumscribed referential domains with a small number of objects. Our study provided clear evidence for spontaneous perspective-taking in a referential communication task using a larger set of objects. Grain of coordination in a preceding non-linguistic task did not affect perspective-taking. However, finer-grained coordination increased the time-locking of comprehension. This result provides the first demonstration that coordination on a non-linguistic task can affect subsequent linguistic processing and lays the foundation for potentially fruitful avenues of new research.

## Methods

### Study design

We used a 2 × 2 × 2 mixed design, with two between-participant variables—coordination (fine-grained vs. coarse-grained) and partner type (computer vs. human), and one within-participant variable—ground (shared vs. privileged). There was no difference in time spent on the puzzle task in the two coordination conditions (*F*(1, 71) = 0.01, *p* = 0.98), which involved the same division of labor and end product. Given previous results on ground effects by Heller et al.^12^: *F*_1_(1,15) = 12.8; *F*_2_(1,15) = 7.4), we expected a large effect size. According to a prior power analysis, a sample size of 52 subjects is needed to obtain a desirable power above 0.8 (effect size *f* = 0.4, calculated with G*Power 3.1^31^).

### Participants

Participants were 75 native speakers of Mandarin Chinese from Peking University (mean age = 23.15, *SD* = 1.49, 54 females), who gave written consent and were paid 35 RMB. Participants were randomly assigned to one of the four between-subject conditions. In the eye-tracking-based referential communication task, data from six participants were excluded due to failing the calibration test or not meeting the “good” standard in the validation test, which requires a worst point error of less than 1.5° and an average error of less than 1.0°. The analyses of task duration time and eye-movements during the puzzle task are reported in Supplementary Table S1.

### Apparatus

An EyeLink-1000 Plus eye tracker (SR Research) measured fixations, sampling at 500 Hz. The puzzle task was implemented using a Python program. Eye-movement data were collected by *Screen-recorder* (SR Research Ltd., Version 1.0.0.1264). The referential communication task was controlled and recorded by *Experiment-Builder* (SR Research Ltd., Version 2.2.1).

### Procedures

#### Manipulation phase: puzzle task

Participants played a two-person puzzle game with a computer partner (participants in the human partner condition believed that they were playing with another person). After reading the game instructions, the participant played two practice rounds (a four-piece puzzle and a 12-piece puzzle). Then the participant and the partner completed the main task with a 48-piece puzzle game.

**Figure 4:**
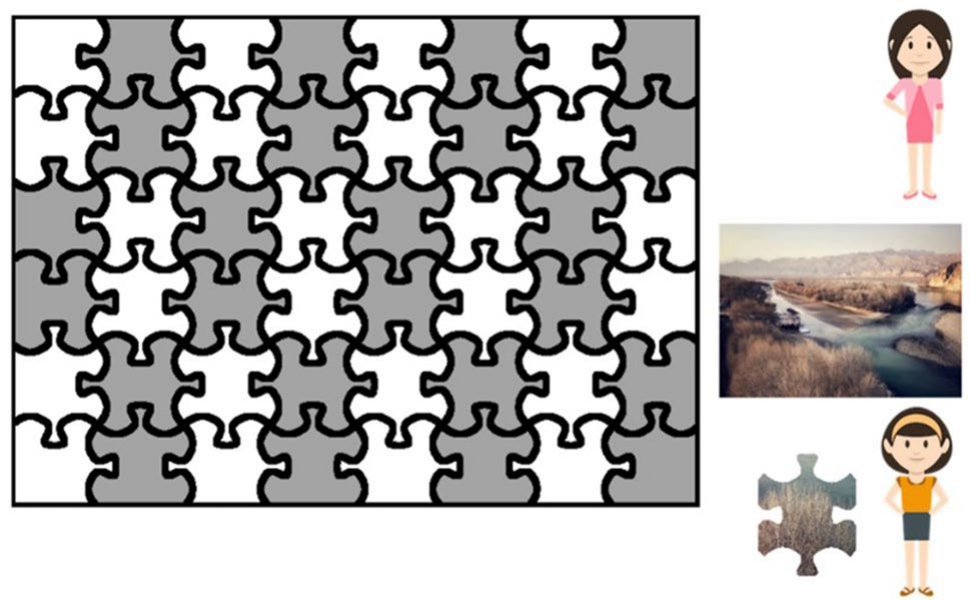
Example display of puzzle task (for female participants). The participant (represented by the avatar at the bottom) completed the white areas and the partner (represented by the avatar at the top) completed the grey areas.

The interface is illustrated in Fig. 4. The participant completed the white areas and the partner completed the grey areas, taking turns placing pieces. The participant first received a piece, displayed beside a same gender avatar. After correctly dragging the piece to the correct place, the next piece for the partner would appear beside the partner’s avatar. The participant waited for the partner to place that piece before receiving the next one.

Pieces and positions to locate them were assigned by algorithms designed for the two coordination conditions. In the fine-grained coordination condition, puzzle pieces were to be placed near their partner’s last-placed piece; in the coarse-grained coordination condition, pieces were to be placed farther away from each other’s last-placed pieces, but close to their own last-placed pieces. The time the computer partner spent placing each piece mimicked the time real participants spent placing similar pieces: Speed increased as the task progressed, and the partner spent less time on corner and edge pieces.

#### Test phase: online referential communication task

**Figure 5:**
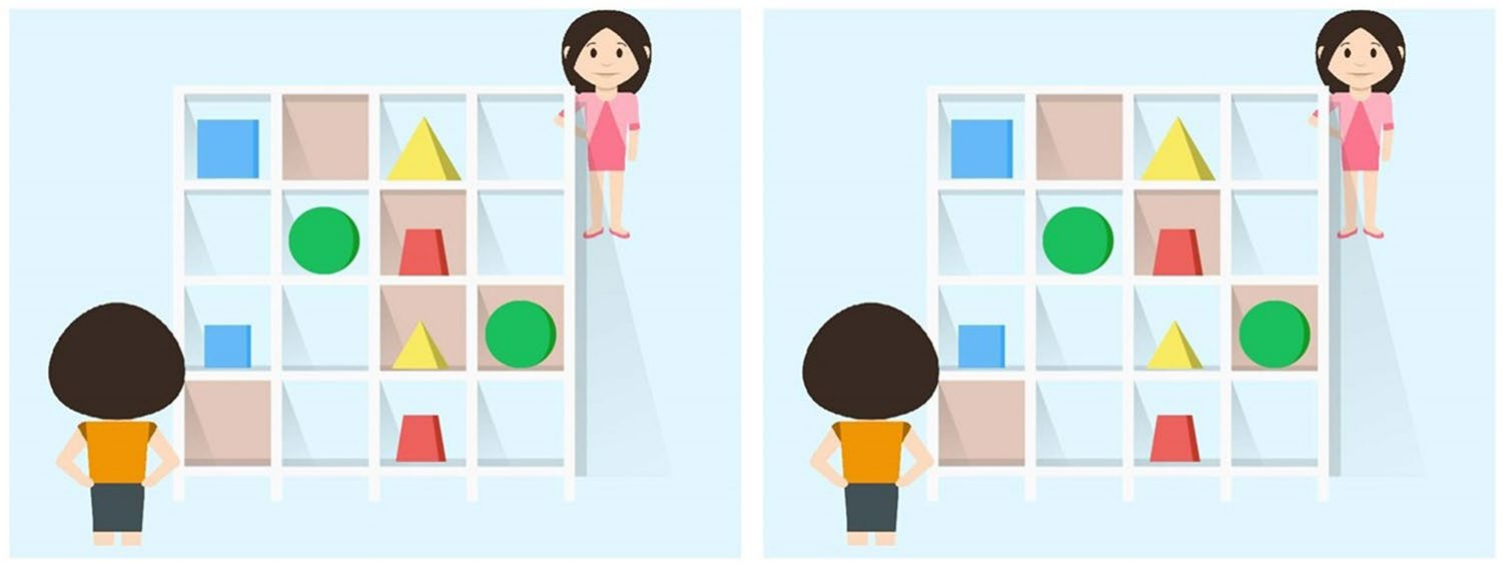
Example displays of two ground conditions in the referential communication task (for female participants). Left panel: privileged-ground condition; right panel: shared-ground condition. Four areas of interests were coded for analysis: target (the big cubic block), competitor (the big triangle block), target-contrast (the small cubic block) and competitor-contrast (the small triangle block). Target position, colors and shapes, and competitor and target-contrast objects were balanced across grid positions.

The task consisted of 16 experimental trials and 16 interspersed filler trials. Each trial paired an auditory sentence and a visual display containing two characters and a shelf with 16 grids (Fig. 5). After five seconds of preview, participants heard pre-recorded instructions in Chinese (voice source: a female native speaker of Chinese) such as the following:

- Chinese: *Qǐng bǎ dàde nà kuài fāngxíng jīmù gěi wǒ.*
- Gloss translation: Please Ba-construction big_MOD DET CL cubic block give me.
- English translation: Please give me the big cubic block.

Participants took the view of the person in front of the shelf. The partner was represented by the character on the other side of the shelf. Shadowed grids are blocked from the view of the girl behind the shelf. There are eight objects in both conditions: In the privileged-ground condition, the competitor-contrast is in the participant’s privileged ground (in the shadowed grid), and hence, not accessible to the speaker; while in the shared-ground condition, the competitor-contrast is visible to both sides (i.e., in common ground).

With the prenominal adjective “big”, listeners assume a referent will have a size contrast. In the privileged-ground condition, listeners could rule out the competitor (the big triangle block in Fig. 5) if they take the speaker’s perspective, as it would be infelicitous for the speaker to use “big” to refer to a big object without a size contrast. In the shared-ground condition, the competitor-contrast is in shared ground and the competitor is a potential referent until the listener hears the shape.

Participants were asked to fill out a questionnaire about how they felt about the tasks and their partners afterwards. The translated questions of the post-test questionnaire and mean scores of each group are summarized in Supplementary Table S2.

#### Partner-type manipulation

The puzzle task began with a screen-based introduction session where both “participants” entered a room representing a test booth. Participants in the computer-partner condition were told they were playing with a computer. In the human-partner condition, participants were told they were playing with another participant in the other booth, and the experimenter acted as checking if the other “participant” was ready via messages at the introduction session before the participants started the puzzle task and in-between the puzzle task and the referential communication task. After completing both tasks, participants were asked if they noticed anything unusual during the experiment. Only one participant in the human-partner condition reported suspicion about the partner, and his data has been removed from analyses.

### Data analysis and coding

We exported proportion of fixations towards the target, competitor and target-contrast in the online referential communication task using the software *Data Viewer* (SR Research Ltd., Version 4.1.1). Multilevel logistic regression models were used to analyze the data. We also analyzed the “target-set”^32^, which includes the target (the big cubic block) and target-contrast (the small cubic block) because listeners typically look at both the target and its contrast following a scalar adjective.

The critical time window for analysis is from 200 ms after the onset of the scalar adjective—“big”/“small” (1 s after the sentence onset and 6 s after the start of the picture on the screen) to 200 ms after the onset of the shape adjective—“cubic”/“sphere”/“triangle”/“trapezium” (2.2 s after the sentence onset and 7.2 s after the start of the picture on the screen). During the 1.2 s critical time period, a time bin of 20 ms was used for analysis. We applied a dummy coding of eye-movement data: The response variable—A *look* was coded as “1” if the subject’s point of gaze was within a specific interest area during this 20ms time bin, and as “0” elsewise. Multilevel logistic regression models were used to analyze the response in function of ground, IA (interest area), coordination and time^33^. The time variable was centered at 0.6 s after onset of the scalar adjective + 200 ms.

We also conducted separate analyses on three 600-ms windows linked to theoretically defined regions in the linguistic utterance (see Supplementary—Window-based Analysis for details). Window-based analyses reduce the effects of multiple correlated observations and provide detailed information about how critical information in specific utterances affected eye-movements. Results from the window-based analyses were consistent with those in the multilevel logistic regression analysis.

### Ethics declarations

Ethics approval was obtained from the Academic Ethical Committee of Peking University. The experiment was performed in accordance with the Declaration of Helsinki. Written informed consent of participation was received from all participants.

### Supplementary Information

Supplementary Information.

## Data Availability

Data and codes that support the findings of this study are openly available in Open Science Framework at https://osf.io/zxp2q/.
