# Supplementary material for: Spontaneous perspective-taking in real-time language comprehension: evidence from eye-movements and grain of coordination
Source: Sci Rep. 2024 Apr 5;14:8031. doi: 10.1038/s41598-024-58699-z (PMC10997771; doi:10.1038/s41598-024-58699-z)
Supplement: Supplementary file 1 — Supplementary Information. [file 41598_2024_58699_MOESM1_ESM.pdf]

Supplementary

Title: Spontaneous perspective-taking in real-time language comprehension: Evidence from eye-movements and grain of coordination

Authors: Yipu Wei, Yingjia Wan*, Michael K. Tanenhaus

1. Window-based Analysis

Eye-movements in the referential communication task were analyzed for three 600 ms time windows. The first window (baseline) captured eye fixations from -400 ms before the beginning of the scalar adjective (e.g., *da* 'big') until 200 ms after it. The second (early) starts from 200 ms after the onset of the scalar adjective and ends at 200 ms after the onset of the classifier (e.g., *kuai* 'piece'). The third (late) contained eye fixations from 200 ms after the onset of the classifier till 200 ms after the onset of the disambiguating shape information (e.g., *fangxing* 'cubic'). We analyzed the proportion of fixations with multilevel linear regression models.

Effects of ground emerged in three different windows. The proportion of fixations to the target in the privileged-ground condition were significantly higher than those in the shared-ground condition in both the early and late windows (early: β = .04, *SE* = .02, *t*(1035) = 2.47, *p* = .01; late: β = .07, *SE* = .02, *t*(1035) = 3.79, *p* < .001), but not in the baseline window (β = .03, *SE* = .02, *t*(1101) = 1.66, *p* = .10). Ground affected looks to the target-set in the same direction during all three windows: more attention was paid to the target-set under the privileged-ground condition in comparison to the shared-ground condition (baseline: β = .04, *SE* = .02, *t*(1034) = 2.07; *p* = .04; early: β = .05, *SE* = .02, *t*(1035) = 3.00; *p* = .003; late: β = .09, *SE* = .02, *t*(1035) = 4.84, *p* < .001). When comparing looks to the competitor and to the target-contrast, there were fewer fixations to the target-contrast than to the competitor (baseline: β = -.03, *SE* = .01, *t*(2152) = -2.09; *p* = .04; early: β = -.07, *SE* = .01, *t*(2220) = -6.35; *p* < .001; late: β = -.12, *SE* = .01, *t*(2220) = -9.39, *p* < .001). However, ground effects interacted with this tendency in the early window and the late window: more looks to the target-contrast were observed in the privileged-ground condition compared to the shared-ground condition (early: β = .04, *SE* = .02, *t*(2220) = 2.30; *p* = .02; late: β = .06, *SE* = .02, *t*(2217) = 3.41, *p* < .001), demonstrating a clear influence of ground throughout the referential processing of scalar adjectives.

The influence of coordination varied across different windows. In the baseline window, the fine-grained coordination group fixated more on the target-contrast compared to the coarse-grained coordination group (β = .04, *SE* = .02, *t*(2152) = 2.18, *p* = .03). Looks to the target-set were not significantly different between the two partner type conditions for the coarse-grained coordination group (β = -.02, *SE* = .02, *t*(66) = -.90, *p* = .37). However, fine-grained coordination participants looked more at the target-set if they believed that they were playing with a real-person partner (β = .10, *SE* = .04, *t*(66) = 2.79; *p* = .01). In the early window, participants from the fine-grained coordination group fixated more on the target-set than the coarse-grained coordination group did (β = .05, *SE* = .02, *t*(68) = 2.08, *p* = .04).

2. Analysis of Puzzle Task

Task Duration Time

We analyzed the average time spent finishing the puzzle task of all 75 participants. The average time was 341.36 seconds (*SD* = 57.00) for the fine-grained coordination group and 341.08 seconds (*SD* = 70.69) for the coarse-grained coordination group. The duration time data was log-transformed analyzed with a linear regression model with two factors: coordination type and partner type. No difference was found between the two coordination groups in terms of the time to finish the puzzle game ($F(1, 72) = .02$, $p = .90$). The type of partner that participants believed they were interacting with did not affect the duration time to finish the task either (human-partner group: mean = 336.26s, *SD* = 47.81; computer-partner group: mean = 346.03s, *SD* = 77.03; $F(1, 72) = .24$, $p = .63$). The results indicate that the manipulations of coordination and partner type did not create differences between conditions in terms of task difficulties.

Eye-movements

With the data from 65 participants whose eye-movements in the puzzle task had been recorded with acceptable quality, we examined the effects of coordination and partner type on each participant's attention to their partners and partners' areas against that to their own. Six interest areas of three sets were included for the analysis, namely partner's puzzle piece, self-puzzle piece, partner's puzzle piece and avatar, self-puzzle piece and avatar, partner's placing area and self-placing area. We computed the partner/self ratio for each set of interest areas. Mean ratios and standard deviations of each condition are presented in Table S1.

Interest areas	Partner type	Partner/Self ratio (SD)	
		Fine-grained coordination	Coarse-grained coordination
Puzzle piece	human	1.40 (.23)	.91 (.23)
	computer	1.77 (.23)	1.50 (.22)
Puzzle piece & avatar	human	1.16 (.14)	.93 (.14)
	computer	1.14 (.14)	1.25 (.13)
Placing region	human	.50 (.04)	.52 (.04)
	computer	.48 (.04)	.43 (.04)

Supplementary Table S1. Partner/self ratio of three sets of interest areas

In a linear regression model, we analyzed partner/self ratios for three sets of interest areas in function of two fixed factors, namely coordination type and partner type, as well as their interactions. Puzzle piece partner/self ratio was lower for conditions where participants were led to believe they were playing with a human partner ($F(1, 61) = 4.48$, $p = .04$). Puzzle piece partner/self ratios differed across different coordination groups with a marginal significance: the fine-grained coordination group paid slightly more attention to their partner's puzzle than the coarse-grained coordination group did ($F(1, 61) = 2.83$, $p = .10$). Coordination type neither influenced the partner/self ratio for the puzzle piece & avatar areas ($F(1, 61) = .20$, $p = .65$), or the partner/self ratio for the placing regions ($F(1, 61) = .16$, $p = .69$). There was no significant effect of partner type either in the analysis for the partner/self ratio of the two interest areas (puzzle piece & avatar: $F(1, 61) = 1.24$, $p = .27$; placing region: $F(1,61) = 1.89$, $p = .17$). No significant interaction effect of coordination or partner type was found in terms of partner/self ratios for any of the three sets of interest areas.

3. Post-test Questionnaire

The nine post-test questions regarding to the feeling of cooperation are presented in Table S2, along with the average ratings in response to these questions of the two coordination groups. A linear regression analysis showed that the fine-grained coordination group scored higher in those questions than the coarse-grained coordination group with a marginal significance ($F(1, 73) = 3.08$, $p = .08$).

The results showed that participants did notice the difference between the fine-grained vs coarse-grained coordination conditions and that difference was related to how their partner behaved in placing the puzzles: In question four, when participants were asked about whether they noticed any connection between their pieces and their partner's pieces in the puzzle game, the fine-grained coordination group rated higher compared to the coarse-grained coordination group ($F(1, 73) = 9.47$, $p = .003$). Most importantly, the manipulation in the prior coordination task did influence participants' sense of collaboration in the follow-up perspective-taking task. In question seven (*In the block-assembly task, did you think your collaboration with your partner went smoothly?*), the mean score of the fine-grained coordination group was also higher than that of the coarse-grained coordination group ($F(1, 73) = 7.40$, $p = .001$).

Questions	Mean (SD) scores for two coordination groups	
(Rating scale: 1-Never ---- 5-Always)	Fine-grained	Coarse-grained
1. In the puzzle game, when a new piece for your partner appeared, did you think of where your partner would place that piece?	2.92 (1.05)	2.77 (.96)
2. In the puzzle game, did you feel like your partner was cooperating with you?	2.86 (1.20)	2.59 (1.46)
3. In the puzzle game, did you feel like you and your partner completed this task together?	3.17 (1.23)	3.08 (1.16)
4. In the puzzle game, did you notice any connection between your pieces and your partner's pieces?	3.44 (.91) **	2.77 (.99)
5. In the block-assembly task, did you take your partner's perspective when thinking of which block your partner asked for?	3.17 (1.58)	3.28 (1.38)
6. In the block-assembly task, when your partner asked for a block, did you take your own perspective first or your partner's perspective first?	3.61 (1.52)	3.21 (1.52)
7. In the block-assembly task, did you think your collaboration with your partner went smoothly?	4.81 (.40) **	4.44 (.72)
8. In both tasks, did you feel that you and your partner had the same goal and shared the same rhythm?	4.06 (.79)	3.85 (.87)
9. In both tasks, did you trust your partner?	4.28 (.78)	4.18 (.85)
Average score	3.59 (.10)*	3.35 (.09)

Supplementary Table S2. Mean scores of fine-grained vs coarse-grained coordination groups in post-test questions

Note: ** Significant difference in mean scores between the fine-grained coordination group and the coarse-grained coordination group. / * Marginally significant difference in mean scores between the two coordination groups.
